# The Impact of Genetic Variation on Drug Response in Adult IBD: A Systematic Review

**DOI:** 10.1002/jgh3.70172

**Published:** 2025-07-22

**Authors:** Masomeh Askari, Shaghayegh Baradaran Ghavami, Nayeralsadat Fatemi, Mahya Haghipanah, Nesa Kazemifard, Hamid Asadzadeh Aghdaei, Makan Cheraghpour, Hamid Mahdizadeh, Shabnam Shahrokh, Mehdi Totonchi

**Affiliations:** ^1^ Basic and Molecular Epidemiology of Gastrointestinal Disorders Research Center Research Institute for Gastroenterology and Liver Diseases, Shahid Beheshti University of Medical Sciences Tehran Iran; ^2^ Department of Stem Cells and Developmental Biology Cell Science Research Center, Royan Institute for Stem Cell Biology and Technology, ACECR Tehran Iran; ^3^ Department of Genetics at Reproductive Biomedicine Research Center Royan Institute for Reproductive Biomedicine, ACECR Tehran Iran

**Keywords:** Crohn's disease, personalized medicine, pharmacogenomics, therapeutic efficacy, ulcerative colitis

## Abstract

**Background:**

A major characterization of inflammatory bowel disease (IBD) is significant heterogeneity in treatment responses. Despite the increase in therapeutic agents, discovering an optimal patient‐level treatment is still a major milestone. This study aims to provide a systematic review of the existing literature on the predictive biomarkers of the response to IBD treatment.

**Methods:**

On April 15, 2025, a literature search was conducted using the PubMed and Scopus databases, as well as a manual search. Controlled trials, case–control studies, cross‐sectional studies, and cohort studies addressing predictive biomarkers for treatment response in adults with IBD were eligible for inclusion. The CASP tool was used to assess the quality of the methodologies employed in the included research. Due to a lack of information and expected heterogeneity, a qualitative study was planned instead of a quantitative one.

**Results:**

Of the 7570 identified articles, 31 met the inclusion criteria. DNA markers were assessed as predictive biomarkers. The majority of studies attempted to predict the response to anti‐TNF drugs. There is significant variability across studies in both the definition of response and the considered biomarkers.

**Conclusions:**

At the moment, no biomarker provides sufficient predictive ability for clinical practice. Thus, we are still in the early stages of the quest for predictive biomarkers, and existing literature is lacking. Future studies should address the large heterogeneity among patients within prospective trials by conducting objective response evaluations. Prediction models are likely to be developed by combining multiple molecular markers from integrated omics levels and clinical characteristics.

## 1 Introduction

Inflammatory bowel disease (IBD) is a chronic condition characterized by inflammation of the gastrointestinal tract, resulting in symptoms such as diarrhea, rectal bleeding, abdominal pain, fatigue, and weight loss [1, 2]. The global incidence of IBD has been increasing over the past decades, and it is estimated that it affects up to 1% of the population worldwide [3]. IBD is divided into two major types, including Crohn's disease (CD) and ulcerative colitis (UC) [4]. The exact cause of IBD remains unclear, but research suggests that it may be caused by improper immune responses to intestinal microbiota, environmental factors, and genetic susceptibility [5].

Epidemiological evidence, including ethnic and racial differences in disease prevalence, familial aggregation, twin studies, and association with other syndromes, has provided substantial insight into the impact of genetic factors on the development of IBD [6, 7]. In recent decades, various studies have been performed to spot the genetic basis of IBD. Furthermore, genome‐wide association studies (GWASs) have been successful at implicating 163 loci, in which approximately 300 potential genes were identified to be associated with IBD risk [8]. The analysis of biological pathways of these genes showed that they play a significant role in maintaining intestinal homeostasis [9]. However, the majority of IBD cases are polygenic, although a fraction of pediatric cases comprises monogenic disorders which have been detected in only approximately 15%–20% of patients with very early onset of IBD [10].

IBD can be treated by either “top‐down” or “accelerated step‐up” strategies. However, it is important to note that IBD is not curable and the final goal of treatment is to induce and prolong the remission phase [11, 12]. In patients with mild‐to‐moderate UC, three groups of drugs including 5‐aminosalicylates [5‐ASA], glucocorticosteroids (CSs), and immunomodulncluding which incorporates thiopurines are used to induce remission [13]. Corticosteroids, antitumor necrosis factor (anti‐TNF) agents (infliximab, adalimumab, and certolizumab pegol), as well as vedolizumab, tofacitinib, and ustekinumab, are suggested for the induction of remission in patients with moderate‐to‐severe disease activity [14]. Although IBD is currently treated by a variety of methods, the significant heterogeneity among patients makes it difficult to select an optimal treatment regimen, and some patients do not respond to any of the existing options. It is estimated that polymorphisms in genes can account for 20%–95% of variability in drug effects [15].

In summary, understanding the genetic factors that contribute to the variability in drug response can provide important insights into the development of personalized treatment strategies for IBD patients and identify potential biomarkers to predict treatment response and guide therapy. This systematic review aims to provide a comprehensive analysis of the existing literature on genetic predictive biomarkers for treatment response in adults with IBD.

## 2 Literature Search Strategy

This systematic review was conducted in accordance with the Preferred Reporting Items for Systematic Reviews and Meta‐Analyses (PRISMA) Statement [16]. A literature search was conducted using the PubMed and Scopus databases, as well as a manual search of Google Scholar, until April 15, 2025. The search strategy included a combination of keywords, as outlined in Data S1. The reference lists of included studies were also hand‐searched to identify any additional relevant studies. Two reviewers independently conducted the literature search, reviewed the titles and abstracts, and consulted to resolve any disagreements.

### 2.1 Inclusion and Exclusion Criteria

Inclusion criteria for the study were (1) randomized controlled trial (RCT), case–control, cross‐sectional, and cohort studies in adult IBD populations; (2) quantitative analysis of interaction between genetic variants and treatment response; (3) the availability of full texts in English. Exclusion criteria included letters, comments, editorials, case reports, conference abstracts, and personal communications.

### 2.2 Data Collection and Assessment of Methodological Quality

Two reviewers extracted the following data from studies that met the inclusion criteria: the name of the first author, year of publication, participant's ethnicity, type of study, number of participants, gene and genetic factor explored, type of protein, comparison of genetic factors and treatment, clinical and statistical outcomes, type of drug and assessment analysis procedure. The methodological quality of the studies was assessed using the Critical Appraisal Skills Programme (CASP) checklist by three reviewers. For RCT studies, CASP contains 11 items divided into three sections, and for cohort studies, it contained 13 items divided into three sections. Conflicts were resolved through discussion or consultation with the fourth author during both the screening process and quality assessment.

## 3 Results

**FIGURE 1:**
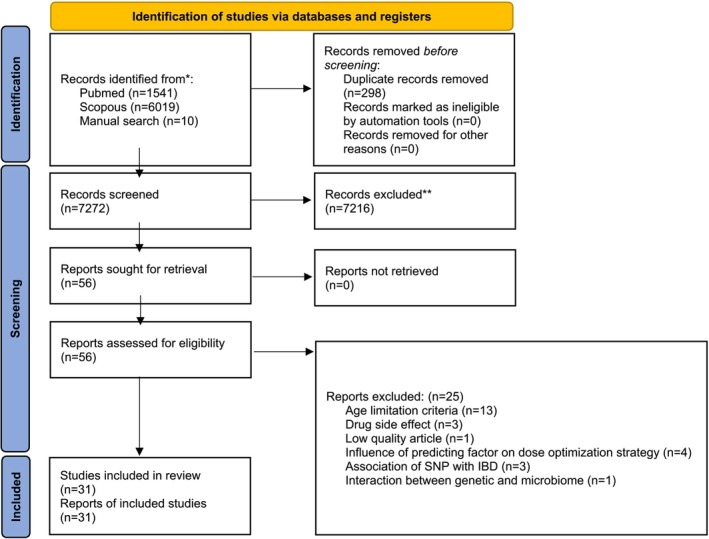
Flow chart for studies included in this systematic review. IBD: inflammatory bowel disease references; SNP: single‐nucleotide polymorphism.

With our search strategy (Figure 1), we aimed to identify all publications covering the impact of genetic variants on therapeutic response in patients with IBD. In total, 7570 articles were found through electronic and manual searches. Based on title and abstract review, 7272 studies were excluded because the publication was not written in English or the study topic did not match our inclusion criteria. In total, 56 articles have been retrieved for full‐text analysis. After reviewing full articles, 25 were excluded for which pediatric samples were used for treatment evaluation, ambushing data, and missing data in clinical drug response. Data from articles with available data on odds ratios (ORs) and 95% confidence intervals (CIs) and genotypes of responder and nonresponder were included (Tables 1, 2, 3).

**TABLE 1:** Included manuscripts for association between polymorphisms and therapy response to Corticosteroids drugs.

Authors and year of publication	Country and institute	Type of study	Population	Genetic factor explored	Clinical response	Type of drug
[17]	Germany	Case–control	276 CD	*HLA‐DRB1 IL‐1ra*	CDAI	Budesonide
[18]	Italy	Case–control	946 IBD	*MDR1*	Endoscopic, radiological and histological criteria	Mesalazine, steroids, immuno suppressives infliximab
[19]	Switzerland	Case–control	185 IBD	*NR3C1*	CDAI	Glucocorticoid
[20]	Germany	Retrospective cohort	185 CD 94 treated with Budesonide	*NOD2*	CDAI	Steroids/immunomedulators, AZA/6‐MP anti‐TNF (infliximab/adalimumab)
[21]	Portugal	Case–control	174 UC 71 treated with corticosteroids	*IL23R*	Reviewing the specific phenotypic chart	Mesalazine (5‐ASA) corticosteroids azathioprine (AZT), infliximab

Abbreviations: CD: Crohn's disease; CDAI: Crohn's disease activity index; IBD: inflammatory bowel disease; UC: ulcerative colitis.

**TABLE 2:** Included manuscripts for association between polymorphisms and therapy response to Immunomodulatory drugs.

Authors and year of publication	Country and Institute	Type of study	Population	Genetic factor explored	Clinical response	Type of drug
[22]	Spain	Case–control	76CD	*ABCB1 (MDR1)*	Harvey–Bradshaw score	Azathioprine
[23]	UK	Cohort	105 CD, 86 UC, 1 IC	*AOX1, XDH, MOCOS*	Red cell TGN levels	Azathioprine
[24]	Israel	Retrospective cohort	156 CD	*Rac1, FASLG*	Montreal classification	Thiopurines
[25]	Germany	Case–control	84 UC	*ABCB1, CYP3A*	Lichtiger index score	Tacrolimus
[26]	Japan	Case–control	61 UC	*CYP3A5, ABCB1*	pDAI, partial disease activity index	Tacrolimus
[27]	Japan	Case–control	29 UC	*CYP3A5*	Mayo score	Tacrolimus
[28]	Japan	Retrospective cohort	87 UC	HLA	Kaplan–Meier method	New therapeutic agents (tacrolimus, infliximab, adalimumab, golimumab, tofacitinib, and vedolizumab)
[18]	Italy	Case–control	946 IBD	*ABCB1 (MDR1)*	Endoscopic, radiological and histological criteria	Mesalazine, steroids, immuno suppressives, infliximab
[20]	Germany	Retrospective cohort	185 CD, 68 treated with azathioprine	*NOD2*	CDAI	Steroids/immunomedulators, AZA/6‐MP anti‐TNF (infliximab/adalimumab)
[21]	Portugal	Case–control	174 UC, 48 treated with azathioprine	*IL23R*	Reviewing the specific phenotypic chart	Mesalazine (5‐ASA) corticosteroids azathioprine (AZT), infliximab

Abbreviations: CD: Crohn's disease; CDAI: Crohn's disease activity index; IBD: inflammatory bowel disease; pDAI: Perianal disease activity index; UC: ulcerative colitis.

**TABLE 3:** Included manuscripts for the association between polymorphisms and therapy response to anti‐TNF drugs.

Authors and year of publication	Ethnicity/country	Type of study	Population	Gene Genetic factor explored	Clinical response	Type of drug
[29]	Belgium	Case–control	226 CD	*TNF*	CDAI	Infliximab
[30]	Belgium	Case–control	200 CD	FCGR3A	CDAI	Infliximab
[18]	Italy	Case–control	478 CD, 468 UC	MDR1	Endoscopic, radiological and histological criteria	Mesalazine, steroids, immuno suppressives, infliximab
[31]	Spanish	Case–control	40 CD	5q31 locus/NOD2	CDAI	Infliximab
[32]	Japan	Case–control	189 CD	*CRP*	CDAI	Infliximab
[33]	Hungary	Case–control	47CD	*ABCB1 (MDR1) ABCG2*	CDAI	Infliximab
[34]	Spain	Case–control	24 CD	*NOD2/CD14/TLR4*	CDAI	Adalimumab
[35]	German	Case–control	90 UC	*IL2/IL21 region IL23R*	CAI	Infliximab
[36]	Spain	Case–control	29CD, 18UC	*IL1B, TNF*	CDAI	Infliximab
[37]	Denmark	Cohort	482 CD, 256 UC	*TLR2, TLR4, TLR5, TLR9, LY96, CD14MAP3K14, SUMO4, NFKBIA NFKB1) TNFA, TNFRSF1A and TNFAIP3 (IL1B, IL4R, IL6IL6R, IL10, IL17AIL23R, IFNG) TGFB1, PTPN22, PPARG NLRP3*	Simple Clinical Three Step Scale (SCTSS)	Anti‐TNF therapy
[21]	Portuguese	Case–control	174 UC (25 treated with infliximab)	*IL23R*	Reviewing the specific phenotypic chart	Mesalazine (5‐ASA) corticosteroids azathioprine (AZT), infliximab
[38]	Spain	Case–control	54 CD, 28UC	*TNF‐a*	Harvey–Bradshaw index (HBI)	Anti‐TNF therapy
[39]	Spain	Case–control	297 CD	*TNFRSF1A, TNFRSF1B*	CDAI	Infliximab
[40]	Slovenia	Case–control	102 CD	*PTGER4, IL27, C11orf30, CCNYI, L13, CASP9*	IBDQ (IBD questionnaire)	Adalimumab
[41]	Greece	Case–control	87 CD	*Survivin*	Harvey‐Bradshaw Index	Infliximab
[42]	Slovenia	Case–control	68 CD	*TF, HFE*	IBDQ	Adalimumab
[43]	Slovenia	Prospective cohort	79 CD	*ATG12, ATG5, NFKB1, NFKBIA, CRP*	IBDQ	Adalimumab
[44]	JAPAN	Case–control	121 CD	*TNF‐α, TNFR1, TNFR2, FCGR2A, FCGR3A*	CDAI	Infliximab
[45]	USA	Cohort	474 IBD	*TNFSF4, TNFSF18, PLIN2, HAUS6, LTF, CCR5, CCRL2, KLHL1, PROX1, RPS6KC1, RORB, TRPM6*	Bowel resection	Adalimumab or infliximab
[46]	Canada	Case–control	152 CD, 110 UC	*HLA Class II Histocompatibility Antigen DQ Alpha Chain*	HBI, Harvey–Bradshaw Index	Infliximab
[47]	Spain	Retrospective cohort	132 CD	*TLR2, TLR4, TLR9, LY96, CD14, MAP3K14, TNFRSF1A, TNFRS1B; FASLG, TNFAIP3 IL1B, IL10, IL6, IL17A*	Endoscopic data	Infliximab
[48]	Polish	Case–control	107 CD	*TNFRSF1A, TNFRSF1B, CASP9, FCGR3A, LTA, TNF, FAS, ADAM17, IL17A, IL6, MMP1, MMP3, S100A8, S100A9, S100A12, TLR2, TLR4, TLR9, CD14, IL23R, IL23, IL1R, and IL1B*	CDAI	Anti‐TNF
[49]	South Brazil	Case–control	16UC, 50CD	*IL‐6*	CDAI and Partial Mayo Index Score	Infliximab or adalimuab

Abbreviations: CD: Crohn's disease; CDAI: Crohn's disease activity index; HBI: Harvey–Bradshaw index; IBD: inflammatory bowel disease; IBDQ: inflammatory bowel disease questionnaire; UC: ulcerative colitis.

Characteristics of the included articles and study quality are provided in Table S1. Thirty‐one studies were focused on adult IBD, of which 23 articles were case–control and seven were cohort studies. Seventeen studies reported associations between polymorphisms and treatment response in CD, five studies focused on UC, and nine articles reported both CD and UC patients. Table 4 gives an overview of genetic biomarkers predictive of the therapeutic response. The gene classification is based on related pathways and therapeutic categories, and the results are discussed in this section. The therapeutic categories considered here comprise conventional treatments such as glucocorticoids and immunomodulatory drugs, anti‐TNF therapy, and combinational therapy. Our analysis sheds light on the roles of various gene pathways in distinct therapeutic categories and offers valuable insights into the potential targets for future research and treatment options.

**TABLE 4:** An overview of genetic biomarkers predictive of the therapeutic response.

Genes	SNP	Sample size	Population	Effect on the response	*p*	Drug	References
5q31 locus	IGR2060a_1	40 CD	Spanish	Association homozygous mutant with lack of response	*p* = 0.024, RR (CI) = 3.88 (1.18–12.8).	Infliximab	[31]
5q31 locus	IGR3081a_1	40 CD	Spanish	Association homozygous mutant with lack of response	*p* = 0.024, RR (CI) = 3.88 (1.18–12.8).	Infliximab	[31]
TLR2	rs3804099	482 CD	Denmark	Association CC and TC genotypes with response to drug	*p* = 0.01, OR = 2.02 95% CI (1.17–3.49)	Anti‐TNF	[37]
TLR2	rs380499	256 UC	Denmark	Association of CC genotype with response to drug	*p* = 0.05, OR = 2.32 95% CI (0.47–11.52)	Anti‐TNF	[37]
TLR2	rs1816702	482 CD	Denmark	Association of CT and TT genotypes with response to drug	*p* = 0.04 OR = 2.02 95% CI (1.04–3.95)	Anti‐TNF	[37]
TLR2	rs11938228	256 UC	Denmark	Association of CA and AA genotypes with nonresponse to drug	*p* = 0.02 OR = 0.49 95% CI (0.26–0.90)	Anti‐TNF	[37]
TLR2	rs4696480	256 UC	Denmark	Association of TT genotype with nonresponse to drug	*p* = 0.04 OR = 0.47 95% CI (0.23–0.95)	Anti‐TNF	[37]
TLR4	rs5030728	482 CD	Denmark	Association of AA genotype with nonresponse to drug	*p* = 0.04, OR = 1.27 95% CI (0.61–2.65)	Anti‐TNF	[37]
TLR4	rs5030728	256 UC	Denmark	Association of AA genotype with response to drug	*p* = 0.02, OR = 2.08 95% CI (0.51–8.46)	Anti‐TNF	[37]
TLR9	rs352139	482 CD	Denmark	Association of AA genotype with nonresponse to drug	*p* = 0.04, OR = 0.38 95% CI (0.16–0.94)	Anti‐TNF	[37]
LY96	rs11465996	482 CD	Denmark	Association GG and GC genotypes with response to drug	*p* = 0.04, OR = 1.97 95% CI = (1.01–2.95)	Anti‐TNF	[37]
LY96	rs11465996	256 UC	Denmark	Association of GG or CG genotype with non‐ response to drug	*p* = 0.01, OR = 0.32 95% CI = (0.14–0.75)	Anti‐TNF	[37]
IL1B	rs4848306	256 UC	Denmark	Association of GG and GA genotypes with response to drug	*p* = 0.04 OR = 2.69 95% CI (1.04–6.94)	Anti‐TNF	[37]
IL6	rs10499563	256 UC	Denmark	Association of CC and TC genotypes with response to drug	*p* = 0.01 OR = 1.61 95% CI (0.94–2.78)	Anti‐TNF	[37]
IL17A	rs2275913	256UC	Denmark	Association of AA and GA genotypes with response to drug	*p* = 0.05 OR = 0.42 95% CI (0.18–1.00)	Anti‐TNF	[37]
TNFRSF1A	rs4149570	738 IBD	Denmark	Association TT genotype with response to drug	*p* = 0.04, CI = (1.03–6.95)	Anti‐TNF	[37]
CD14	rs2569190	256 UC	Denmark	Association of GA or AA genotype with non‐ response to drug	*p* = 0.05, OR = 3.19 95% CI 0.95–16.78.	Anti‐TNF	[37]
TNFRSF1B	rs3397	297 CD	Spanish	Association of CC genotype with remission	*p* = 0.04, OR = 0.54 95% CI 0.30–0.98	Infliximab	[39]
TNFRSF1B	rs1061624	297 CD	Spanish	Association of AA genotype with remission	*p* = 0.006, OR = 2.84 95% CI (1.25–6.59)	Infliximab	[39]
CASP9	rs4645983	102 CD	Slovenia	Association CC genotype with poor response	*p* = 2.44E‐02	Adalimumab	[40]
ATXN2L	rs8049439	102 CD	Slovenia	Association CC genotype with poor response	*p* = 2.96E‐02	Adalimumab	[40]
CCNY	rs12777960	102 CD	Slovenia	Association CC genotype with good response	*p* = 1.56E‐02; OR: 3.26; 95% CI: 1.27–8.38	Adalimumab	[40]
IL13	rs1295686	102 CD	Slovenia	Association AA genotype with good response	*p* = 6.07E‐03	Adalimumab	[40]
HFE	rs2071303	68 CD	Slovenia	Association AA genotype with lack response	*p* = 0.009, OR = 4.832, 95% CI = 1.519–15.367	Adalimumab	[42]
ATG5	rs9373839	79 CD	Slovenia	Association TT genotype with lack response	*p* = 1.13·10^−04^	Adalimumab	[43]
CRP	rs1130864	79 CD	Slovenia	Association TT genotype with lack response	*p* = 4.09·10^−05^	Adalimumab	[43]
TNFSF4	rs116724455	474 IBD	American	Association CT genotype with positive response	*p* = 4.79E‐08, OR: 19.9 [4.57–86.6]	Anti‐TNF	[45]
HLADQA1*05	rs2097432	262 IBD	Canadian	Association AA genotype with positive response	*p* = 0.001, HR = 2.34, 95% CI = 1.41–3.88	Infliximab	[46]
TLR2	rs1816702	132 CD	Spanish	Association CC genotype with better long‐response	*p* = 0.049, HR, 0.128; 95% CI, 0.02–0.99	Infliximab	[47]
TLR2	rs3804099	132 CD	Spanish	Association TT genotype with improved long‐response	*p* = 0.023, HR, 0.039; 95% CI, 0.18–0.88	Infliximab	[47]
TNFRSF1B	rs1061624	132 CD	Spanish	Association GG genotype with lack log‐response	*p* = 0.030, HR,0.041; 95% CI, 0.18–0.92	Infliximab	[47]
Rac1	rs34932801	156CD	Israel	Association of GC genotype with lower response	OR = 0.26; 95% CI = 0.08–0.91, *p* = 0.036	Thiopurine	[24]
ABCB1	rs1128503	84UC	Germany	Association TT genotype with short‐term remission.	OR = 1.29 (90% CI) = (1.08–1.54), *p* = 0.030	Tacrolimus	[25]
ABCB1	rs1045642	84UC	Germany	Association of TT genotype with short‐term remission.	OR = 1.09, (90% CI) = (1.02–1.17), *p* = 0.030	Tacrolimus	[25]
ABCB1	rs2032582	84UC	Germany	Association of TT genotype with short‐term remission.	OR = 1.29, (90% CI) = (1.03–1.6), *p* = 0.030	Tacrolimus	[25]
ABCB1	rs1128503	61UC	Japan	Association of CC and CT genotypes with higher response	P response = 0.004, P remission = *p* = 0.002	Tacrolimus	[26]
HLA	rs117506082	87 UC	Japan	Association of AA genotype and long‐term outcomes	HR: 2.11, (95% CI) = (1.07–4.14), *p* = 0.03	New therapeutic agents (tacrolimus, infliximab, adalimumab, golimumab, tofacitinib, and vedolizumab)	[28]
HLA‐DR	HLA‐DRB1	276 CD	Germany	Association of heterozygous and homozygous mutant of HLA‐DRB1 with failed to response	*p* = 0.00067	Budesonide	[17]
IL23R	C2370A	71 UC	Portugal	Association of AA genotype with negative response	*p* = 0.02	Corticosteroids	[21]

Abbreviations: CD: Crohn's disease; UC: ulcerative colitis.

### 3.1 Conventional IBD Treatments

In this systematic review, two studies have specifically examined the influence of genetic variants on glucocorticoid response. Both studies utilized the Crohn's disease activity index (CDAI) as the measure of clinical response. The CDAI values decreased in both studies, indicating a positive clinical response to treatment (Table 1). Moreover, several studies have been conducted to investigate the potential correlation between polymorphisms in genes associated with immunomodulatory drugs and the efficacy of clinical therapy. There were six studies included in this systematic review that investigated the contribution of genetic variants to immunomodulatory drugs response, three of them in patients with IBD who took Azathioprine (AZA), and three in patients with UC who took Tacrolimus (Table 2). The results of these studies are categorized into five cellular pathways: transport of small molecules, adaptive immunity, glucocorticoid receptors (GRs), pattern recognition receptors (PRRs) signaling pathway, and metabolic pathway.

#### 3.1.1 Transport of Small Molecules

The *ABCB1* (*MDR1*) gene encodes for a transmembrane efflux pump that plays a pivotal role in drug disposition and response by actively pumping a plethora of xenobiotics out of cells. The efflux mechanism is, particularly, relevant in various physiological barriers, such as the gastrointestinal tract, where it contributes to drug absorption, distribution, metabolism, and excretion. The *ABCB1* efflux pump is also associated with multidrug resistance, a phenomenon that limits the efficacy of chemotherapy in cancer patients. The association between two polymorphisms in *ABCB1* (rs2032582 and rs1045642) and AZA response was examined in a study of 982 Italian patients with IBD. No significant association of the two polymorphisms with AZA response was found [18]. However, it was demonstrated that, in patients without response to AZA, the frequency of the TT genotype of rs2032582 (17.65%) was higher than that in the responders (7.14%). Of interest, among those without a response to AZA, patients with the TT genotype of rs1045642 had a higher frequency (17.54%) compared with clinical‐remission‐achieved CD patients (4.76%) [22]. Another study of 396 CD patients revealed that the two polymorphisms in the *ABCB1*gene (rs1045642 and rs2032582) were not related to glucocorticoid response [18]. In 89 UC patients treated with Immunomodulatory treatment, three *ABCB1* single‐nucleotide polymorphisms (SNPs) (rs1128503; rs2032582; or rs1045642) had notably greater rates of success in reaching short‐term remission relative to heterozygous individuals and those with the reference sequence [25]. In line with this evidence, in a study on 61 tacrolimus‐treated UC patients from Japan. The *ABCB1* rs1128503 was significantly associated with therapeutic efficacy. In comparison with the TT group (*n* = 20), the response rate and remission rate of the rs1128503 CC + CT groups (*n* = 41) were considerably higher (73% vs. 35% with *p* = 0.004 and 63% vs. 20% with *p* = 0.002, respectively) [26].

#### 3.1.2 Adaptive Immunity

Adaptive immunity‐related genes, particularly, the human leukocyte antigen (HLA) molecules, are pivotal in the pathogenesis of IBD and its response to therapy. The HLA molecules are involved in regulating the adaptive immune response by presenting antigens to T cells. A genetic alteration in the *HLA‐DR8* was associated with budesonide resistance in CD patients [17]. Based on a multicenter study, the AA genotype of *IL23R* C2370A was associated with a negative response in 71 patients with UC treated with corticosteroids (*p* = 0.02) [21]. Furthermore, an association of polymorphism in *IL23R* with AZA response was performed in A study on 48 patients treated with AZA, and it was found that the GG genotype for *IL23R*_G9T was more likely to respond to AZA (*p* = 0.05) [21].

#### 3.1.3 GRs

Glucocorticoids can bind to the cytoplasmic GR as ligands and inhibit T cell activation and cytokine secretion. A study by Mwinyi et al. explored the *NR3C1* genetic variants in response to steroid therapy in 181 IBD patients. This study found no significant association between variation in the *NR3C1* gene and glucocorticoid therapy success [19].

#### 3.1.4 PRRs Signaling Pathway

Pathogen recognition receptors (PRRs) (such as nucleotide oligomerization domain [NOD]‐like receptors [NLRs] and Toll‐like receptors [TLRs]) are a class of cell receptors in charge of recognizing pathogen‐associated molecular patterns (PAMPs) and play a crucial role in the proper function of the innate immune system. In a study on *NOD2* variants and glucocorticoid therapy response, there was a higher prevalence of *NOD2* mutation carriers among those with a systemic steroid requirement (8.9%) than those without (WT 1) (*p* = 0.0086) and those with local‐steroid resistance (14.9% vs. 3.5%; *p* = 0.001) in UC patients [20]. Furthermore, *NOD2* variants and AZA responses were evaluated in a cohort study. It was proven that 65% of patients with *NOD2* WT status went into remission under treatment with AZA/6‐MP, whereas 34% of patients with *NOD2* WT status were refractory to treatment with AZA/6‐MP. Eighty‐eight percent of patients with *NOD2* variants went into remission under treatment with AZA/6‐MP, and 12% of patients with *NOD2* variants were refractory to treatment with AZA/6‐MP [20].

#### 3.1.5 Metabolic Pathways

A multicenter study was conducted to assess whether genetic polymorphism in *AOX1*, *XDH*, and *MOCOS* is associated with AZA treatment outcomes in 192 IBD patients. After sequencing 12 polymorphisms, It was shown that only a SNP in *AOX1* c.3404A>G (Asn1135Ser, rs55754655) predicted the lack of AZA response (*p* = 0.035, OR 2.54, 95% CI 1.06–6.13) [23]. Given the ability of thiopurines to induce T‐cell apoptosis by modulation of Rac1 activation, nine polymorphisms in the *Rac1* were screened. It was shown that the *Rac1* SNP rs34932801 heterozygote genotype GC was associated with a lower response rate compared with the wild‐type GG genotype (46% vs. 76%; OR ¼ 0.26; 95% CI, 0.08–0.91; *p* = 0.036) [24].

Eighty‐nine UC patients from Germany were investigated to determine the contribution of *CYP3A5* variants to treatment outcomes. According to the results, they found no significant association between cYP3a5*3 and treatment response (*p* = 0.030) [25]. In line with this evidence, the influence of *CYP3A5* polymorphism on the efficacy of tacrolimus treatment was investigated in 61 UC patients from Japan. Based on the results, no association was found between the CYP3A5 genotypes and therapeutic efficacy [26]. Moreover, according to the results of a study that examined the correlation between *CYP3A5* genetic polymorphisms and therapeutic response or colectomy rate in 29 Japanese UC patients, there was no correlation between these factors [27].

### 3.2 Anti‐TNF Therapy (IFX and ADA)

Despite the widespread use of tumor necrosis factor (TNF) inhibitors for the treatment of severe cases of CD and UC, a significant proportion of patients, as estimated through clinical research, exhibit an ineffective response to this form of therapy, with an estimated rate between 20% and 40% [39]. In this systematic review based on defined criteria, 23 studies were included, of which 14 studies assessed the contribution of genetic factors to TNF response in CD samples; however, only two studies analyzed responders versus nonresponders in UC samples, and 15 studies used both CD/UC samples. The assessment of clinical response in these studies was based on various criteria, which are summarized in Table 3. An overview of predictive genetic biomarkers for anti‐TNF response is provided in Table 4. Our findings suggest that the genes involved in the response to anti‐TNF therapy can be categorized into various cellular pathways. These pathways include the transport of small molecules, phagocytosis, the complement system, cytokine signaling, the TNF signaling pathway, PRRs signaling pathway, cell division and death, and adaptive immune response.

#### 3.2.1 Transport of Small Molecules

IBD has been observed to exhibit an association with a diverse range of proteins, including transporters, which play a pivotal role in determining the pathogenesis and treatment response of the disease. These transporters have been identified as key players in the absorption and secretion of ions and solutes across the epithelial barrier of the intestinal mucosa. In this regard, two studies have focused on the multidrug resistance (*MDR1*) gene, which codes for *MDR1* (*ABCB1*), a member of the ATP‐binding cassette (ABC) transporters. In a large cohort of 948 Italian IBD patients, Palmieri et al. evaluated the genotype of two polymorphisms of *MDR1* (rs2032582 and rs1045642) but found no significant association with clinical response [18]. Another study investigated the relationship between rs2032582 and rs1045642 in *ABCB1* and rs2231137 in *ABCG2* and clinical response to Infliximab in both CD and UC patients. Both *MDR1* and *ABCG2* variants failed to predict response to steroid or infliximab in CD or the need for surgery in either UC or CD [33]). Correlation between the SNP rs2071303 in gene *HFE* and response to anti‐TNF treatment with ADA was also investigated in CD patients. Following a 20‐week treatment period, 84.4% of individuals with the AG or GG genotypes showed positive responses to treatment with ADA relative to 52.8% of individuals with the AA genotype (*p* = 0.009, OR = 4.832, 95% CI = 1.519–15.367) [42].

#### 3.2.2 Phagocytosis

Phagocytosis is a complex process for eliminating pathogens and cell debris. It is usually followed by inflammatory pathway activation and associated with IBD pathogenesis. A total of 200 CD patients with FCGR3A‐158 V/F genotypes were assessed for their response to infliximab. No significant difference was found between the two groups (80.0% responses in V/V patients vs. 63.0% responses in V/F and F/F patients; *p* = 0.11) [30]. In addition, in a group of 121 Japanese CD patients, the relationships between therapeutic response to IFX during long‐term maintenance treatment and genetic polymorphisms were investigated, but no association was found for *FCGR2A* A>G (rs1801274) and FCGR3A T>G (rs396991) [44]. As the important role of autophagy‐related genes in IBD, adalimumab therapy was found significantly associated with genotypes CC and CT of rs9373839 in *ATG5* (*p* = 1.13·10–04) [43].

#### 3.2.3 Complement System

Previous studies report that a positive clinical response to infliximab has been associated with an elevated C‐reactive protein (CRP) level before treatment. Hence, in a study from Willot et al., there was no significant association between the studied *CRP* gene polymorphisms (−717G/A, 1444C/T, and CRP 4A/G) and the clinical response to infliximab in 189 Crohn's disease cases [32]. However, it was demonstrated that genotypes CC and CT (*p* = 4.09·10–05) of rs1130864 in the *CRP* gene were associated with a positive response to ADA [43].

#### 3.2.4 Cytokine Signaling Pathway

A large number of cell types that coordinate cytokine‐mediated intercellular communication were associated with IBD, and several studies confirmed that genetic variations in cytokine networks not only caused IBD but also affect drug responses. A study was conducted to evaluate the effect of *IL23R* variants and *IL2* / *IL21* variants on the response to IFX in 90 patients with UC. In addition, the Colitis activity index (CAI) and markers of inflammation were measured during IFX induction therapy. The genotypes of four variants in the *IL2*/*IL21* region (rs13151961, rs13119723, rs6822844, and rs6840978) and 10 *IL23R* SNPs (rs1004819, rs10889677, rs11209032, rs2201841, and rs1495965) (Increased IBD susceptibility); rs7517847, rs10489629, rs11465804, rs11209026, and rs1343151 (decreased IBD susceptibility) were genotyped. No significant differences were found between IFX responders and nonresponders regarding the distribution of the minor allele when comparing the single *IL23R* SNPs and *IL2*/*IL21* region SNPs. However, homozygous carriers of IBD risk‐increasing *IL23R* variants were more likely to respond to IFX than homozygous carriers of IBD risk‐decreasing *IL23R* variants (74.1 vs. 34.6%; *p* = 0.001) [35]. Lacruz‐Guzmán et al. for the first time evaluated the pharmacogenetic role of rs1143634 IL1B in CD and UC patients. In 18 UC patients showing partial or no response, 87.5% (7/8) of patients were CC carriers, whereas 60% (6/10) of UC responders were CC carriers; this difference did not reach statistical significance (*p* = 0.196). In 29 CD patients, after 14 weeks of infliximab treatment, none of the genotypes (CC vs. CT, TT *p* = 0.12) were related to a particular infliximab response [36]. Another study was also conducted to assess the interaction between genetic variants in the *IL23R* gene and infliximab response in 25 UC patients. There was no association between *IL23R* genetic variants and infliximab response [21]. The effect of variations in *IL1B*, *IL6*, and *IL17A* on TNF response in 256 patients with UC was investigated. It was shown that not only patients with UC who carried GG or GA genotypes of *IL1B* (rs4848306) (*p* = 0.04) showed a better outcome, but also the TC or CC genotype of *IL6* rs10499563 (*p* = 0.01) was associated with clinical response. There was an increased risk of the lack of response with homozygous *IL17A* 197G4A genotype (rs2275913) (*p* = 0.05). However, no association was found between the *IL6* genetic variants and TNF‐α inhibitor response (rs1800795, rs1800796) [49]. In the *IL13* gene, the rs1295686 SNP showed a correlation with IBDQ. Compared with patients with AG or GG genotypes, delta IBDQ after a long‐term treatment was higher in patients with AA genotype (*p* = 6.07E‐03) [40]. In addition, the genotypes of the IBD5 locus were analyzed in the Spanish CD cohort, and the association between homozygous mutant genotypes with CD patients lacking response was confirmed [31].

#### 3.2.5 TNF Signaling Pathway

It is known that cytokine TNF alpha has a critical role in inflammation. In this regard, some research has focused on the impact of variations in these genes on TNF therapy. The effect of two SNPs at the TNF‐a promoter gene (rs361525 and rs1800629) on anti‐TNF‐a response was investigated in a number of IBD patients from Spain. No significant difference was found in allele and genotype frequencies of rs361525 regarding responses to TNF‐a inhibitor (IFX or ADA) treatment. They found a higher incidence of the –GA genotype of rs1800629 in the nonresponders to anti‐TNF treatment with respect to responders' patients (*P*_c_ < 0.05) [38].

By considering 297 CD patients, no association between rs767455 located in *TNFRSF1A* and infliximab response was found [39]. Nevertheless, the homozygous variant genotype of *TNFRSF1A*–609G4T (rs4149570) (OR_adj_: 2.39, 95% CI: 1.03–5.57, *p* = 0.04) was correlated with a beneficial response among 738 patients with CD and UC [37]. Furthermore, another study examined the association of the variant in *TNFRSF1B* with drug response. It was shown that two *TNFRSF1B* SNPs were significantly associated with remission including the minor genotype (CC) rs3397 (*p* = 0.006, OR = 3.19 95% CI 0.95–16.78) and minor genotype (AA) rs1061624 (*p* = 0.006), OR = 2.84, 95% CI (1.25–6.59) [39]. In contrast with this evidence, 132 CD patients were genotyped, and it was reported that in the multivariate *TNFRSF1B* model (rs1061624), the predictive ability of long‐term response to IFX was considerably greater for AA and GA genotypes than the GG genotype [47]. As reported by Matsuoka et al., no statistically significant difference was observed in terms of the relationship between therapeutic response to IFX maintenance therapy and known genetic polymorphisms (*TNFR1*) A>G [rs767455], *TNFR2* G>A [rs976881], *TNFR2* T>G [rs1061622], *TNF‐α‐238* G>A (rs361525), *TNF‐α‐308* G>A (rs1800629), *TNF‐α‐ 857* C>T (rs1799724) [44]. In 474 patients with IBD, Wang et al. examined the clinical significance via the correlation of rs116724455 genotype status in the *TNFSF4* gene with various clinical outcomes and characteristics. The correlation between CT genotype and anti‐TNF response was significant (*p* = 4.79E‐08, OR: 19.9 [4.57–86.6]) [45].

#### 3.2.6 PRRs Signaling Pathway

The *NOD2/CARD15* gene is known as the first identified CD susceptibility gene. The *NOD2* SNPs have also been associated with different clinical forms of the disease in different populations. However, the *NOD2* gene variants (rs2066845, rs2066844, and rs2066847) were not associated with ADA response in Spanish CD patients [34].

Several lines of evidence indicated that TLRs and TLR‐activated signaling pathways are implicated in both IBD pathogenesis and treatment efficacy. In CD patients, the heterozygous genotype of *TLR4* (rs5030728) *p* = 0.01 and both the homozygous and the heterozygous variant genotypes of *TLR2* (rs3804099) *p* = 0.01, and (rs1816702) *p* = 0.04, and *LY96* (rs11465996) (*p* = 0.04) were associated with a positive response. However, the homozygous variant genotypes of *TLR4* (rs5030728) (*p* = 0.04) and *TLR9* (rs352139) (*p* = 0.04) were associated with nonresponse [37]. A correlation was found between two variants of *TLR2* and an enhanced response to IFX. Compared with the TT genotype, rs1816702 C in homozygosis predicted a better response to IFX (HR, 0.128; 95% CI, 0.02–0.99; *p* = 0.049). In addition, over a decade‐long follow‐up, the response to IFX was maintained in individuals with the TT genotype relative to patients with the CC genotype (HR, 0.039; 95% CI, 0.18–0.88; *p* = 0.023) [47].

In UC patients, the homozygous variant genotype of *TLR4* (rs5030728) (*p* = 0.02), and the homozygous variant genotype of *TLR2* (rs3804099) (*p* = 0.05) were associated with beneficial responses among patients with UC. Nonetheless, the homozygous variant genotype of *TLR2* (rs4696480) (*p* = 0.04) and both the homozygous and the heterozygous variant genotypes of *TLR2* (rs11938228) (*p* = 0.02), *LY96* (rs11465996) (*p* = 0.01), *CD14* (rs2569190) (*p* = 0.04) were associated with nonresponse in UC patients [37]. Although multiple studies highlighted the role of *TLRs* variants in drug response in IBD. No significant association was found between *CD14* (rs2569190) and *TLR4* (rs4986790) in response to adalimumab [34].

#### 3.2.7 Cell Division and Death

A number of studies have been conducted to examine the relationship between recently discovered genes that play a role in cell division and death, and their impact on the effectiveness of TNF therapy in treating IBD. It has been demonstrated that rs4645983 in the *CASP9* gene is associated with response to treatment with adalimumab. A positive response to treatment was found in patients with the CT or TT genotypes when compared with those with the CC genotypes (*p* = 2.44E−02). In addition, the effect of rs12777960 located on gene *CCNY* on treatment response was investigated. The CC genotype was associated with better treatment response to ADA on the basis of IBDQ criteria. Among patients with the CC genotype, 77.8% responded to treatment after 4 weeks, compared with 51% of those with the AA or AC genotype (*p* = 1.56E‐02; OR: 3.26; 95% CI: 1.27–8.38) [40].

#### 3.2.8 Adaptive Immune Response

As previously indicated, susceptibility to IBD is partially genetically determined and the HLA molecules and their variants play a central role in the immune response. Therefore, HLA typing and their genetic variants could be a valuable tool for predicting the clinical outcome of IBD patients and customizing their treatment plans accordingly. In a recent study conducted, it was demonstrated that HLADQA1*05 A>G (rs2097432) variant carriers were at an increased risk of infliximab loss of response (adjusted HR = 2.34, 95% CI = 1.41–3.88, *p* = 0.001) [46].

### 3.3 Combination Therapy

A cohort study [28] was conducted to evaluate the long‐term prognosis of Japanese patients with UC who were treated with new therapeutic agents such as tacrolimus, infliximab, adalimumab, golimumab, tofacitinib, and vedolizumab. The study also looked at the association between prognosis and genetic susceptibility to UC. The results showed that a shorter time from diagnosis to initiation of treatment with new therapeutic agents was found to be a risk factor for colectomy. Additionally, the study identified this SNP rs117506082 in 87 patients and found significant differences in disease location and time from diagnosis to colectomy between the groups with the GG + GA and AA genotype. The AA genotype of this SNP was associated with a shorter time to surgery and increased use of new therapeutic agents (HR =2.11, 95% CI = 1.07–4.14, *p* = 0.031).

## 4 Discussion

The success rate of current treatments for IBD remains relatively limited, with a maximum efficacy of 50% [50]. However, identifying genetic variations that are linked to drug response in IBD patients has the potential to enhance the selection of the most appropriate treatment type and drug dose [51].

Investigations into the genetic determinants of drug response in IBD are faced with significant challenges. One of the major difficulties lies in the complex pathogenesis of IBD, which has been implicated in a wide range of dysregulated pathways that are crucial for maintaining intestinal homeostasis [5]. Furthermore, the interactions between the immune system, genome, intestinal microbiota, and environmental stimuli play a significant role in the development and progression of IBD [52]. Given the multifactorial nature of IBD pathogenesis, it is unlikely that a single gene or a narrow gene spectrum within a specific pathway would provide a complete explanation for the tendency to respond to a particular targeted treatment [53]. Our study has classified related genes into cellular pathways and has analyzed their response to three distinct therapy regimens. Specifically, metabolic pathway genes were found to be unique to traditional therapy response, while genes involved in cell division, death, and autophagy were assigned to anti‐TNF therapy. These observations suggest that the cellular pathways activated by each therapy regimen may differ and have implications for the development of personalized treatment plans.

The reproducibility of results represents the second issue. There are often contrasting results even in research efforts examining the same polymorphism. This variability could be attributed to several factors, including the small sample sizes in some of the studies or the diversity of ethnic groups involved in the research (e.g., Danish vs. Spanish populations) [37, 47]. On the other hand, there is a lack of standardization in the reporting of results across studies, with each analysis being conducted differently and adjusting for varying factors or not adjusting at all. Our systematic review has highlighted the role of genetics as a determinant of drug response in IBD. However, due to the heterogeneity of existing literature, it is difficult to accurately quantify the extent of this relationship, particularly, in relation to anti‐TNFα biological drugs, which comprise the majority of the research.

Despite the fact that research works have reported both positive and negative results, several genetic variants in identified IBD genes (*TLR4, MDR1, and CRP*) may predict the efficiency of anti‐TNFα biological drugs [48]. The TLR gene family, in particular, has been the subject of extensive investigation and has been linked to infliximab therapy [9]. The dysregulation or dysfunction of TLR expression and activation, as well as the presence of genetic polymorphisms in *TLR* genes, is believed to contribute to the development and progression of IBD [50]. The association of *TLR2, TLR4*, and *TLR9* with response to anti‐TNF therapy in IBD has been established despite the fact that TLRs function as the hub of immune responses to gut microbes, leading to the triggering of IBD [54]. After reviewing the results of 23 studies based on TNF therapy, this systematic review showed that *TLR2* variants (such as rs3804099 and rs1816702) in IBD patients are promising markers for the prediction of response to anti‐TNF therapy response.

Other genes identified in systematic studies to contribute to the efficacy of treatment included the TNF receptor family, which comprises 19 ligands and 29 receptors [55]. There are two ways for the contribution of the members of the TNF superfamily to IBD pathogenesis: (i) altering the intestinal epithelium integrity and inducing apoptosis in enterocytes (FasL, TNF, TWEAK, and TRAIL), and/or (ii) increasing the pro‐inflammatory activity of mucosa that infiltrate mononuclear cells (TL1A, TNF, TWEAK, LIGHT, and potentially FasL) and changing the activity of regulatory T cells and regulatory macrophages [56]. Multiple research projects indicated that genetic variants in *TNFR* not only lead to IBD but also impact the outcome of anti‐TNF therapy [57]. According to our review, the link between *TNFRB1* variants and positive drug response in IBD has been established in two separate studies [39, 47].

Additionally, the relationship between genetic variants in *ABCB1* and immunomodulatory response has been investigated in multiple studies. The gene encodes for P‐glycoprotein (P‐gp), a transmembrane protein that plays a crucial role in bowel diseases [57]. Research has shown that *ABCB1* polymorphisms are associated with drug tolerance, metabolism of chemotherapeutic agents, and disease susceptibility [58]. Our systematic review revealed that four studies have explored the link between different polymorphisms in *ABCB1* and response to immunomodulatory drugs. Association with drug response was not significant in the populations of Italy and Spain [18, 22] while there was an association between rs1128503 and drug response in German and Japanese populations [25, 27].

In our methodological systematic review, a comprehensive search was conducted to include all relevant studies pertaining to the research questions. However, the study is subject to several biases and limitations. One such limitation is the use of a candidate gene approach, which did not meet the genome‐wide significance threshold. Additionally, the study did not consider environmental factors that could potentially impact genetic susceptibility. Moreover, several studies examining the association between polymorphisms and biological response were not included in the analysis due to the presence of heterogeneity in the characterization of the population, exposure, and outcome. This heterogeneity precluded the ability to perform a meta‐analysis to determine the extent to which genetic factors contribute to drug response in IBD.

In conclusion, we managed to detect a genetic component contributing to treatment response among all the considered studies. The integration of genetic analysis into clinical research has the potential to greatly enhance the prediction of treatment responses and pave the way for the development of personalized medications. However, it is important to note that there is still a lack of information on the pharmacogenetics of drug therapy in IBD, and further research is required to fully realize the benefits of personalized medications in clinical practice.

## Ethics Statement

The authors have nothing to report.

## Consent

The authors have nothing to report.

## Conflicts of Interest

The authors declare no conflicts of interest.

## Supporting information


**Data S1.** Search strategies.

**Table S1.** Characteristics of the included articles and study.

## Data Availability

Data sharing is not applicable to this article as no datasets were generated or analyzed during the current study.
