# Supplementary material for: The Impact of Genetic Variation on Drug Response in Adult IBD: A Systematic Review
Source: JGH Open. 2025 Jul 22;9(7):e70172. doi: 10.1002/jgh3.70172 (PMC12281211; doi:10.1002/jgh3.70172)
Supplement: Supplementary file 1 — Data S1. Search strategies. [file JGH3-9-e70172-s002.docx]

Search strategies

Part 1:

- IBD (MeSH) OR CD(MeSH) OR CU(MeSH) OR Crohn's Enteritis OR Regional Enteritis OR Crohn's Disease OR Crohns Disease OR Inflammatory Bowel Disease 1 OR Enteritis, Granulomatous OR Granulomatous Enteritis OR Enteritis, Regional OR Ileocolitis OR Colitis, Granulomatous OR Granulomatous Colitis OR Ileitis, Terminal OR Terminal Ileitis OR Ileitis, Regional OR Regional Ileitides OR Regional Ileitis OR Idiopathic Proctocolitis OR Ulcerative Colitis OR Colitis Gravis OR Inflammatory Bowel Disease, Ulcerative Colitis Type OR Bowel Diseases, Inflammatory OR Unclassified Colitis OR Indeterminate Colitis. Non-specific inflammation of the GASTROINTESTINAL TRACT OR Chronic inflammatory bowel diseases OR Very early onset inflammatory bowel disease.

Part 2:

- Genetic variant (MeSH) OR Biomarker (MeSH) OR Genetic susceptibility (MeSH) OR Mutation (MeSH) OR Disease susceptibility (MeSH) OR Risk Factor (MeSH) OR Pharmacogenomic Variant OR Variant, Pharmacogenomic OR Variants, Pharmacogenomic OR Pharmacokinetic Genetic Variants OR Genetic Variant, Pharmacokinetic OR Genetic Variants, Pharmacokinetic OR Pharmacokinetic Genetic Variant OR Pharmacogenetic Variants OR Pharmacogenetic Variant OR Variant, Pharmacogenetic OR Variants, Pharmacogenetic OR Factor, Risk OR Risk Factor OR Health Correlates OR Correlates, Health OR Risk Scores OR Risk Score OR Score, Risk OR Risk Factor Scores OR Risk Factor Score OR Score, Risk Factor OR Population at Risk OR Populations at Risk OR Diathesis OR Diatheses OR Susceptibility, Disease OR Disease Susceptibilities OR Susceptibilities, Disease OR Genetic Susceptibility OR Genetic Susceptibilities OR Susceptibilities, Genetic OR Susceptibility, Genetic OR Genetic Predisposition OR Genetic Predispositions OR Predispositions, Genetic OR Predisposition, Genetic OR Marker, Biological OR Biological Marker OR Biologic Marker OR Marker, Biologic OR Biological Markers OR Biologic Markers OR Markers, Biologic OR Biomarker OR Markers, Biological OR Markers, Immunologic OR Immune Markers OR Markers, Immune OR Marker, Immunologic OR Immunologic Markers OR Immune Marker OR Marker, Immune OR Immunologic Marker OR Serum Markers OR Markers, Serum OR Marker, Serum OR Serum Marker OR Surrogate Endpoints OR Endpoints, Surrogate OR Surrogate End Point OR End Point, Surrogate OR Surrogate End Points OR End Points, Surrogate OR Surrogate Endpoint OR Endpoint, Surrogate OR Markers, Clinical OR Clinical Markers OR Clinical Marker OR Marker, Clinical OR Viral Markers OR Markers, Viral OR Viral Marker OR Marker, Viral OR Biochemical Marker OR Markers, Biochemical OR Marker, Biochemical OR Biochemical Markers OR Markers, Laboratory OR Laboratory Markers OR Laboratory Marker OR Marker, Laboratory Surrogate OR Markers Markers, Surrogate OR Marker, Surrogate.

Part 3:

- Prognosis [Mesh] OR Severity of Illness Index [Mesh] OR Precision Medicine [Mesh] OR Prognoses OR Prognostic Factors OR Factor, Prognostic OR Factors, Prognostic OR Prognostic Factor OR Illness Index Severities OR Illness Index Severity OR Disease Severity OR Disease Severities OR Severity, Disease OR Biological signature OR Medicine, Precision OR Personalized Medicine OR Medicine, Personalized OR Individualized Medicine OR Medicine, Individualized OR P Health OR P-Health OR Predictive Medicine OR Medicine, Predictive OR Theranostics OR Theranostic OR Clinical outcomes OR Pathogenesis OR Patient stratification.

Part 4:

- Part 1 and Part2 and Part 3.
- Abstract
- English language.
