# Supplementary material for: The Impact of Genetic Variation on Drug Response in Adult IBD: A Systematic Review
Source: JGH Open. 2025 Jul 22;9(7):e70172. doi: 10.1002/jgh3.70172 (PMC12281211; doi:10.1002/jgh3.70172)
Supplement: Supplementary file 2 — Table S1. Characteristics of the included articles and study. [file JGH3-9-e70172-s001.docx]

Supplementary Table 1. Characteristics of the included articles and study.

| **Assessment analysis procedure** | **Type of drug** | **Comparison** | **Gene** | **Genetic factor explored** | **Population** | **Type of study** | **Country** | **Authors and year of publication** |
| --- | --- | --- | --- | --- | --- | --- | --- | --- |
| Reverse hybridization polymerase chain reaction for HLA-DRB1 Polymerase chain reaction and electrophoresis for IL-1ra polymorphism | Budesonide | An association analysis of HLA-DRB1 genotypes and IL-1ra gene polymorphism with treatment response to budesonide | HLA-DRB1 IL-1ra | Genotypes 1–16 86-bp region: allele 1 (four repeats), allele 2 (two repeats), Allele 3 (five repeats), Allele 4 (three repeats) | 276 CD | Case-control | Germany | Gelbmann et al., 2001 |
| polymerase chain reaction | Infliximab | Comparison of genotype frequencies between responders and non-responders | TNF | TNF -308 SBP substitution TNF1 TNF2 | 226 CD | Case-control | Belgium | Louis et al., 2002 |
| Allele-speciﬁc polymerase chain reaction a | received inﬂiximab received inﬂiximab received inﬂiximab Infliximab | association between the FCGR3A-158 polymorphism and biological and clinical responses to inﬂiximab in Crohn’s disease association between the FCGR3A-158 polymorphism and biological and clinical responses to inﬂiximab in Crohn’s disease association between the FCGR3A-158 polymorphism and biological and clinical responses to inﬂiximab in Crohn’s disease Association analysis between the FCGR3A-158 polymorphism and biological and clinical responses to infliximab in Crohn’s disease | FCGR3A | FCGR3A-158V/F | 200 CD | Case-control | Belgium | Louis et al., 2004 |
| Polymerase chain reaction | Mesalazine, steroids, Immunosuppressives and infliximab. | Investigation of genetic polymorphisms in MDR1 gene and response to drugs | ABCB1 (MDR1) | rs1045642 rs2032582 | 946 IBD Patients 450 healthy control | Case-control | Italy | Palmieri et al., 2005 |
| TaqMan assay Allele-specific PCR | Infliximab | Association analysis of genotype with response to infliximab | 5q31 locus NOD2 | IGR2060a_1 IGR3081a_1 (rs2066847 rs2066844 rs2076756) | 40 CD | Case-control | Spanish | Urcelay et al, 2005 |
| Polymerase chain reaction PCR-restriction fragment length polymorphism (RFLP) | Infliximab | Evaluate the association between CRP gene polymorphisms and response to infliximab | CRP | CRP -717A/G CRP 1444T/C CRP 4A/G | 189 CD | Case-control | Japan | Willot et al., 2006 |
| Polymerase chain reaction (PCR Genotyping was performed using the Light Cycler. | Infliximab | Association between the ABCG2 and MDR1 polymorphisms and the response to infliximab therapy | ABCB1 (MDR1) ABCG2 | rs2032582 rs1045642 rs2231137 rs2231142 | 47CD | Case-control | Hungary | S. Fischer et al., 2007 |
| Analyzed by SYBR Green assay, Real time PCR | Azathioprine | To investigate the contribution of *MDR1*) gene to the efficacy of azathioprine in inducing remission in patients with CD. | ABCB1 (MDR1) | rs2032582 rs1045642 | 76CD | Case-control | Spain | Mendoza et al., 2007 |
| Real-time PCR, TaqMan SNP genotyping assay | Azathioprine (AZA) | To assess whether genetic polymorphism in AOX1, XDH and MOCOS is associated with AZA treatment outcome in IBD. | AOX1 XDH MOCOS | rs55754655 rs4407290, rs17323225, rs17011368, rs2295475, rs1884725, rs207440 rs623053, rs59445, rs1057251 | 105 CD 86 UC 1 IC | Cohort Study | UK | Smith et al., 2009 |
| Polymerase chain reaction followed by restriction fragment length polymorphism (PCR-RFLP) | Adalimumab | Evaluation the association between selected genes and response to adalimumab treatment | NOD2 CD14 TLR4 | rs2066844 rs2066845 rs2066847 rs2569190 rs4986790 | 24 CD | Case-control | Spain | Barreiro-de Acosta et al., 2010 |
| Genotyping included amplification by a PCR and melting curve analysis | Infliximab | Assessment of variants in IL23R and in the IL2 / IL21region in response to IFX in UC | IL2/IL21 region IL23R gene | rs13151961 rs13119723 rs6822844 rs6840978 rs1004819, rs2201841, rs7517847, rs1495965, rs11209032, rs10489629, rs11465804, rs11209026 rs1343151, rs10889677 | 90 UC | Case-control | German | Jürgens et al., 2010 |
| Polymerase chain reaction followed by Sequencing | steroid therapy | Analysis of NR3C1 haplotypes and variants in relation to steroid therapy outcome. | NR3C1 | Haplotype GR_4merged, GR_2 merged, GR_3 merged, E22E/R23K, N363S, rs4986593, rs6188, rs258750, rs10482704, | 185 IBD | Case-control | Switzerland | Mwinyi et al., 2010 |
| Genotyping assays by MassARRAY Compact system | Tacrolimus | The assessment of genetic variants in CYP3A4/5 and ABCB1 to Tac response in patients with UC | ABCB1 CYP3A | rs1128503; rs2032582; rs1045642 CYP3A4*1B CYP3A5*3 | 84 UC | Case-control | Germany | Herrlinger et al., 2011 |
| Polymerase chain reaction (PCR) -sanger sequencing | Steroids/ immunomedulators , AZA/6-MP anti-TNF (infliximab/adalimumab) | Investigation of drug response in patients with NOD2 variant. | NOD2 | rs2066844 rs2066845 rs2066847 | 185 CD | Retrospective cohort | Germany | Niess et al., 2012 |
| KBioscience chemistry kit, Polymerase chain reaction (PCR) | Infliximab | Identification of clinical and genetic predictive marker of TNF and/or IL1β as surrogate markers of infliximab response. | IL1B TNF | rs1143634 rs1799964 rs4647198 rs1800630 rs1799724 rs4248158 rs4987086 rs4248159 rs2736195 rs4248160 rs3093548 rs4248163 rs55994001 rs55634887 rs1800750 rs1800629 rs361525 | 29CD 18UC | Case -control | Spain | Lacruz-Guzmán et al., 2013 |
| TaqMan SNP Genotyping Assays | Thiopurines | Assessment of polymorphisms in Rac1 and other apoptosis-related genes with response to thiopurine therapy | ac1 FASLG Caspase 9 | rs4939 rs34932801 rs35144878 rs763110 rs4645983 | 156 CD | Retrospective cohort | Israel | Koifman et al., 2013 |
| Allele-Specific Polymerase chain reaction (KASP), an end-point PCR technology, | Anti-TNF therapy | Associations between functional polymorphisms in the NFkB signaling pathway and response to anti-TNF treatment | TLR2, TLR4, TLR5, TLR9, LY96, CD14, MAP3K14, SUMO4, NFKBIA NFKB1) TNFA, TNFRSF1A and TNFAIP3 (IL1B, IL4R, IL6 IL6R, IL10, IL17A IL23R, IFNG) TGFB1, PTPN22, PPARG NLRP3 | rs4696480, rs1816702, rs11938228, rs3804099 rs12377632, rs5030728, rs1554973 rs5744168 (rs187084, rs352139), (rs11465996), (rs2569190), (rs7222094), (rs237025), (rs696, rs17103265), (rs28362491), (rs1800629, rs1800630, rs1799724, rs361525), (rs4149570), (rs6927172), IL1B (IL-1b) (rs1143623, rs4848306, rs1143627), (rs4251961), IL4R (rs1805010), IL6 (rs10499563), (rs4537545), (rs1800872, rs3024505), (rs2275913), (rs11209026), (rs2430561), (rs1800469), (rs2476601), (rs1801282 (rs4612666). | 482 CD 256 UC | cohort | Denmark | Bank et al., 2014 |
| Real-time PCR using Taqman probes | Mesalazine (5-ASA) Azathioprine (AZT), (infliximab) | Identify clinical and genetic (SNPs in IL23R) predictor of response to therapy in patients with UC | IL23R | G1142A, C2370A, G43045A, G9T | 174 UC | Case -control | Portuga | Cravo et al., 2014 |
| PCR-SSOP using a microbeads luminex assay | Anti-TNF therapy | To investigate the role that these SNPs of TNF-a promoter gene play in the risk of IBDs in a Spanish population and the individual response to anti-TNF-a treatment. | TNF-a promoter gene | rs361525 rs1800629 | 54 CD 28UC | Case-control | Spain | López-Hernández et al., 2014 |
| Genotyping by Taqman technology | Infliximab | Investigation of role of variants in IFX response in CD patients | TNFRSF1A TNFRSF1B | rs767455 rs1061622, rs1061624, rs3397 | 297 CD | Case -control | Spain | Medrano et al., 2014 |
| PCR followed by restriction fragment length polymorphism | Adalimumab | Correlation between SNPs & response to ADA | PTGER4 IL27 C11orf30 CCNY IL13 CASP9 | rs10512734 rs8049439 rs7927894 rs12777960 rs1295686 rs4645983 | 102 CD | Case -control | Slovenia | Koder et al., 2015 |
| SNP genotyping assays on real-time PCR | Adalimumab | Associations between selected SNPs in genes HFE and TF and response to anti-TNF treatment with ADA in Slovenian CD patients | TF HFE | rs1799852 rs2071303 | 68 CD | Case -control | Slovenia | Repnik et al., 2016 |
| Genotyping by Taqman technology | Adalimumab | Investigation of association of SNPs in genes with the response to ADA therapy measured with IBDQ | ATG12, ATG5, NFKB1, NFKBIA, CRP | rs26538 (rs9373839, rs510432) (rs3774934, rs4648011, rs13117745) rs696 rs1205, rs1130864 | 79 CD | prospective cohort study | Slovenia | Dezelak et al., 2016 |
| Genotyping by Taqman technology | Tacrolimus | Investigate the influence of both CYP3A5 and ABCB1 polymorphisms on the efficacy of tacrolimus in ulcerative colitis treatment under the tight dose-adjusting strategy | CYP3A5 ABCB1 | rs776746 rs1128503 rs2032582 rs1045642 | 61 UC | Case -control | Japan | Onodera et al., 2017 |
| Genotyping by real-time pcr (taq man) | Tacrolimus | Investigation of adverse events,C/D ratio and clinical outcome in UC patients | CYP3A5 | (rs776746) | 29 UC | Case -control | Japan | Asada et al., 2017 |
| TaqMan SNP Genotyping assay | Infliximab | Investigation of predictive factors associated with the response to IFX in long-term maintenance treatment | TNF-α TNFR1 TNFR2 FCGR2A FCGR3A | rs361525, rs1800629, rs1799724 rs767455 rs976881, rs1061622 rs1801274 rs396991 | 121 CD | Case -control | JAPAN | Matsuoka et al., 2018 |
| GWAS Study | Adalimumab 0r Infliximab | Identification of explored model integrating clinical and genetic predictors of anti-TNF response in patients with IBD. | TNFSF4, TNFSF18, PLIN2, HAUS6, LTF, CCR5, CCRL2, KLHL1, PROX1, RPS6KC1 RORB, TRPM6 | rs116724455 rs2228416 rs762787 rs9572250 rs144256942 rs523781 | 474 IBD | cohort | USA | Wang et al,2019 |
| Real-time taq man | Infliximab | Investigation the association between the rs2097432 and risk of infliximab loss of response | HLA Class II Histocompatibility Antigen DQ Alpha Chain | rs2097432 | 152 CD 110 UC | Case-control | Canada | Wilson et al., 2020 |
| Real time PCR | Infliximab | Identification of polymorphisms associated with long-term response to IFX in patients with CD | TLR2, TLR4, TLR9, LY96, CD14, MAP3K14, TNFRSF1A, TNFRS1B; FASLG, TNFAIP3 IL1B, IL10, IL6, IL17A | rs1816702, rs3804099, rs5030728, rs352139, rs11465996 rs2569190 rs7222094 rs4149570, rs767455 rs1061622, rs1061624, rs3397, rs1800629, rs361525, rs6927172, rs763110, rs1800872, rs3024505 rs4848306, rs10499563, rs2275913 | 132 CD | Retrospective cohort | Spain | Salvador-Martín et al., 2019 |
| long-range PCR libraries and the next generation sequencing (NGS) method | Anti-TNF | Investigation of pharmacogenetic biomarker of anti-TNF therapy in CD Patients. | TNFRSF1A, TNFRSF1B, CASP9, FCGR3A, LTA, TNF, FAS, ADAM17, IL17A, IL6, MMP1, MMP3, S100A8, S100A9, S100A12, TLR2, TLR4, TLR9, CD14, IL23R, IL23, IL1R, IL1B) FCGR3A IL1R TNFRSF1B IL1B FAS | rs7539036, rs6672453, rs373184583, rs12128686, rs2041747, rs5746053, rs1071676, rs1143639, rs1143637, rs1143634, rs7896789 | 107 CD | Case-control | polish | Walczak et al., 2020 |
| Real-time PCR (Taq man) | Infliximab, Adalimuab | Investigation the association between the genetic variants and the outcome of TNF-α inhibitors treatment | IL-6 | rs1800795 rs1800796 | 16UC, 50CD | Case -control | South Brazil | Gonçalves et al., 2021 |

CD: Crohn's Disease, IBD: Inflammatory Bowel Disease, UC: Ulcerative Colitis
